# Supplementary material for: Serotonin/5-HT1A Signaling in the Neurovascular Unit Regulates Endothelial CLDN5 Expression
Source: Int J Mol Sci. 2020 Dec 29;22(1):254. doi: 10.3390/ijms22010254 (PMC7795278; doi:10.3390/ijms22010254)
Supplement: Supplementary file 1 [file ijms-22-00254-s001.pdf]

Figure S1

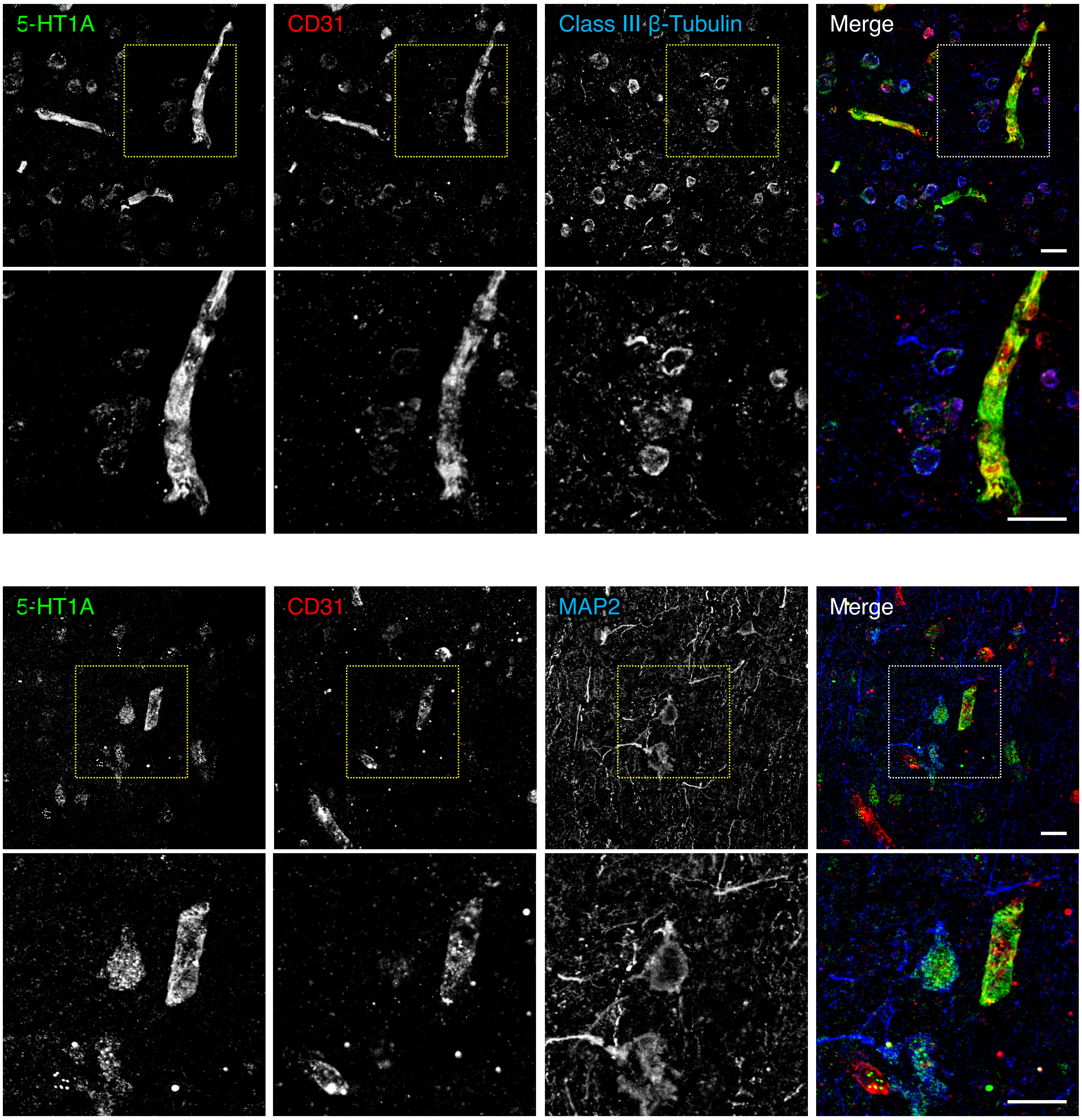

Figure S1. 5-HT1A receptor is expressed in microvascular endothelial and perivascular cells of normal human PFC. The normal PFC gray matter, which was obtained from a different donor than that in Figure 1, was stained for 5-HT1A and CD31 together with either class III β-tubulin or MAP2. Scale bars, 20 μm.

Supplementary table 1. Antibodies

Antigen	Host	M/P	Dilution	Source	Identifier
5-HT1A	Rabbit	Polyclonal	1:100	GeneTex	N3C1
αSMA	Mouse	Monoclonal	1:200	Dako	M0851
CD31	Mouse	Monoclonal	1:100	Dako	M0823
CD34	Mouse	Monoclonal	1:101	Dako	M7165
Class III β-Tubulin	Chicken	Polyclonal	1:100	abcam	ab41489
CLDN5	Mouse	Monoclonal	1:100	ThermoFisher SCIENTIFIC	35-2500
CLDN5	Rabbit	Polyclonal	1:200	IBL	18855
GFAP	Mouse	Monoclonal	1:100	Sigma-Aldrich	G3893
MAP2	Chicken	Polyclonal	1:100	abcam	ab5392
PDGFRβ	Goat	Polyclonal	1:100	R&D systems	AF385
pPKAα/β/γ	Rabbit	Polyclonal	1:500	Santa Cruz	32968
ZO-1	Rabbit	Polyclonal	1:200	ThermoFisher SCIENTIFIC	61-7300
Rabbit IgG (Alexa 488)	Donkey	Polyclonal	1:200	Jackson ImmunoResearch	711-545-152
Chicken IgG (Cy3)	Goat	Polyclonal	1:200	abcam	ab97145
Goat IgG (Cy3)	Donkey	Polyclonal	1:200	Jackson ImmunoResearch	705-165-147
Mouse IgG (Cy3)	Donkey	Polyclonal	1:201	Jackson ImmunoResearch	715-165-150
Mouse IgG (Alexa 647)	Donkey	Polyclonal	1:200	Jackson ImmunoResearch	211-605-109
